# The scope of childhood cancer in South Africa: A response to ‘Childhood cancers in a section of the South African private health sector – Analysis of medicines claims data’

**DOI:** 10.4102/hsag.v26i0.1672

**Published:** 2021-11-29

**Authors:** Jaques van Heerden, Mariana Kruger

**Affiliations:** 1 Department of Paediatrics and Child Health, Faculty of Medicine and Health Sciences, Stellenbosch University, Cape Town, South Africa; 2 Department of Paediatric Haematology and Oncology, Antwerp University Hospital, Antwerp, Belgium

**Keywords:** childhood cancer, medical policy, medical aid claims, incidence, oncology

## Abstract

Childhood cancer is an under resourced medical field that is emerging as a great healthcare concern in low- and middle-income countries such as South Africa. Therefore, reporting data in this field that may inform policymakers should be representative of the subject matter. This article aims to discuss why medicines claims as an indicator for incidence, as per an article published in 2020, is not representative of childhood malignancies in the South African setting. Literature to support the commentary were sourced using Pubmed, Google scholar, and data presented by members of the South African Children’s Cancer Study Group (SACCSG). Private medical aid coverage in South Africa between 2002 and 2018 varied between 15.5% and 18.2%. Of these, 9.5% were children under 18 years and 3.5% were under the age of six. Only 13.5% of children were treated in private paediatric oncology units during 2015. The limitations in the study were the variable medical aid coverage, the disproportionate age representation, and lack of reliable indicators for measurement and calculation of incidence. Utilising one medicines claims data base to evaluate the incidence of childhood cancer in South Africa is not representative and cannot inform policy.

**Contribution:**

This article highlights the importance of accurate registration of childhood cancer diagnoses, especially when data and conclusions based on these results inform policy. The study highlights the limitations of extrapolating general conclusions based on data representing only a small sector of the childhood cancer landscape in South Africa.

## Introduction

The incidence of childhood malignancies is increasing globally (Gupta et al. 2014). This is because of the improved awareness, diagnostic capabilities, and the increased quality of reporting of incidences (Gupta et al. 2014). Nonetheless, independent from the resource setting, distinguishing the non-specific early symptoms of childhood cancer from more common childhood diseases remains challenging even with the increased quality of medical systems (Stones 2010:314).

The lack of high-quality data has been cited as a reason for inaccurate reporting of the incidence of childhood cancers in sub-Saharan Africa and South Africa (Erdmann et al. 2015:2628; Magrath et al. 2013:104). It is not only because of the absence of registries, but that registers are dependent on healthcare workers and administrators to include data into the registries (Stefan et al. 2015:939). These registries have quality controls to ensure accurate reporting. This includes screening for double registrations and the use of different identifiers for the same patient (Stefan et al. 2015:939). Other sources to describe possible incidences in childhood malignancies are available, but lack the same standards necessary for representative information.

The medicines claims of the South African private health sector was analysed by Otoo et al. (2020:a1382) based on a database of a single South African Pharmaceutical Benefit Management (PBM) company. The market share of the PBM was not included but covered 1.8 million beneficiaries. During a 10-year period between 2008 and 2017, only 173 new cases of childhood cancers were identified in the database. The article thus concluded that the incidence of childhood cancer in a part of the private medical sector was decreasing.

As stated in the article, data are used to develop cancer control strategies, evaluate changes in disease burden, and implement policies (Otoo et al. 2020:a1382). Most importantly, data informs the policy. Therefore, an accurate representation and conclusions based on data are of great importance.

## Aim

The aim of this commentary is to discuss why medicines claims as an indicator for incidence is not representative of childhood malignancies regardless of the sector of the population in the South African setting.

## Comments

### Limitations in the Otoo et al. (2020) study

The original study partially acknowledged the limitations that the study was based on a single PBM database analysis, that there are discrepancies in incidences and the diverse way in which medical accounts are paid, but still maintained that the data could be used for policy purposes (Otoo et al. 2020:a1382). In addition, two important limitations of the study are that it does not indicate if the patients were newly diagnosed during the study period and the ‘medicine claims’ on which data extraction was based, were never defined.

Oncology patients are treated over years. If the patient does not die from the disease, the treatment period and follow up stretches over years. During this period, many cancers and non-cancer related medical claims can be submitted. Therefore, a single medical claim cannot indicate the date of diagnosis. The date of diagnosis is the indicator that determines in which year a particular patient is counted as part of the incidence (Steliarova-Foucher et al. 2017). Furthermore, it is not clear whether the patients were still in treatment. A ‘medicine claim’ could have been made for a non-cancer diagnosis in a patient out of treatment. Hence, the importance of a definition for ‘medicine claims’.

In South Africa there are numerous PBM companies, of which one of the largest only administrates just over half of the private medical aid funds registered with the Council of Medical Schemes of South Africa (2020) and Mediscore Pharmaceutical Benefits Management (2021) thereby excluding the four largest medical aid schemes in the country (Council of Medical Schemes 2017:186). It is therefore, possible that the study represents less than half of the children covered by private health insurance, that have been treated or are being treated for a childhood malignancy, and have made a claim against the fund for a cancer related or unrelated treatment.

### Heterogeneous medical systems, sector of management and remuneration

**FIGURE 1:**
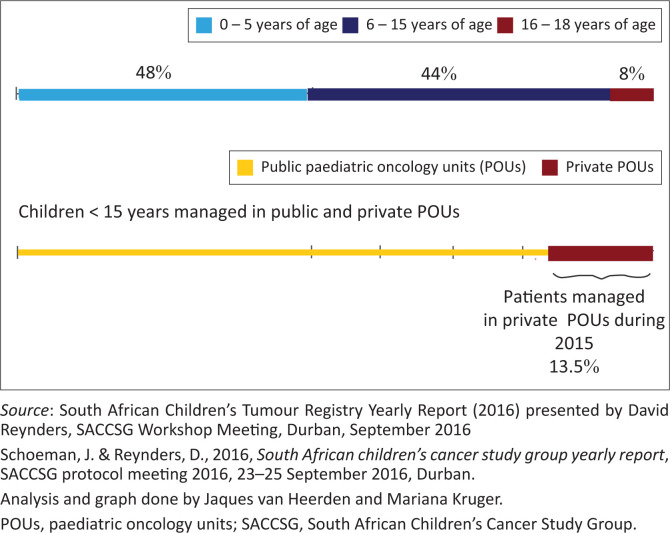
Number of children treated by members of the South African Children’s Cancer Study Group (SACCSG) according to age at diagnosis in 2015.

The South African medical system is heterogeneous comprising both the private and public sector (Coovadia et al. 2009:817). In the public sector, children under the age 6 year are treated for free. The remuneration in the public sector is based on the income per household, but also manages patients with private medical funding (Coovadia et al. 2009:817). The private sector is remunerated by both private medical funding and non-medical aid payments (Coovadia et al. 2009:817). Based on the national household surveys from StatsSA between 2003 and 2019, the percentage of the population covered by private medical aid between 2002 and 2017 had the lowest coverage in 2004 (15.5%) and the highest coverage in 2013 (18.2%) (see Table 1) (StatsSA 2018:26). Therefore, if all the PBM’s data could be analysed, it would still represent less than a fifth of health data of the South African population. It is reported that more older people are covered by medical aid funds, whilst younger adults with young families are covered to a lesser degree by private medical funding (Omotoso & Koch 2017:575; Van den Heever 2012:S5). Thus, when calculating the percentage of children covered by medical aid in 2017, it is probable that it is less than 16.9% reported by StatSA. Based on data from the South African Children’s Study Group, in 2015 only 13.5% of children were treated in private paediatric oncology units (POUs) in South Africa (see Figure 1) (Schoeman & Reynders 2016). The private medical aid coverage for the under 18-years age group has been reported to be 9.5% and for the under 6 years age group 3.5% (Omotoso & Koch 2017:575; Van den Heever 2012:S5). It is possible that the percentage of medical aid claims that are because of childhood cancer is far lower than reported in the Otoo et al. (2020) study. Considering the representation of the database (less than 50% of medical aid claims) used in the study, the conclusions are only applicable to less than 10% of the childhood malignancies in South Africa.

**TABLE 1:** The percentage of the South African population covered by private medical aid between 2002 and 2017.

Indicator	Year									
	2002	2004	2008	2010	2012	2013	2014	2015	2016	2017
**Percentage of population covered by private medical aid**	15.9	15.5	16.3	17.7	17.6	18.2	17.7	17.1	17.1	16.9
**Percentage of children in South Africa**	38.0	36.4	36.6	35.8	35.4	35.1	34.8	34.7	34.6	35.0

*Source*:Statistics South Africa (StatsSA), 2018, *General Household Survey 2018*, pp. 26–27, Statistics South Africa, Pretoria, viewed 16 April 2021, from http://www.statssa.gov.za/publications/P0318/P03182018.pdf

Analysis by Jaques van Heerden and Mariana Kruger.

Beneficiaries do change private medical aid funding for various reasons (Omotoso & Koch 2017:575; Van den Heever 2012). The implication is that patient (encrypted) numbers may change. Although not the only data collected, this was the primary indicator used in their study (Otoo et al. 2020:a1382). Cancer treatment is done over years, which limits the age of a patients as a reliable identifier. Referrals between private oncology units do occur and families relocate for several reasons. Referrals may take place because of the need for specialised expertise such as bone marrow transplantation. Utilising the prescriber’s postal code would further contribute a confounder to the study. In a large dataset, these confounders may have a limited impact on the results, but in the Otoo et al. (2020) study, confounders may contribute to multiple representation of the same patient in the dataset. Thus, an overestimation of the incidence.

### Distribution of paediatric oncology units

**FIGURE 2:**
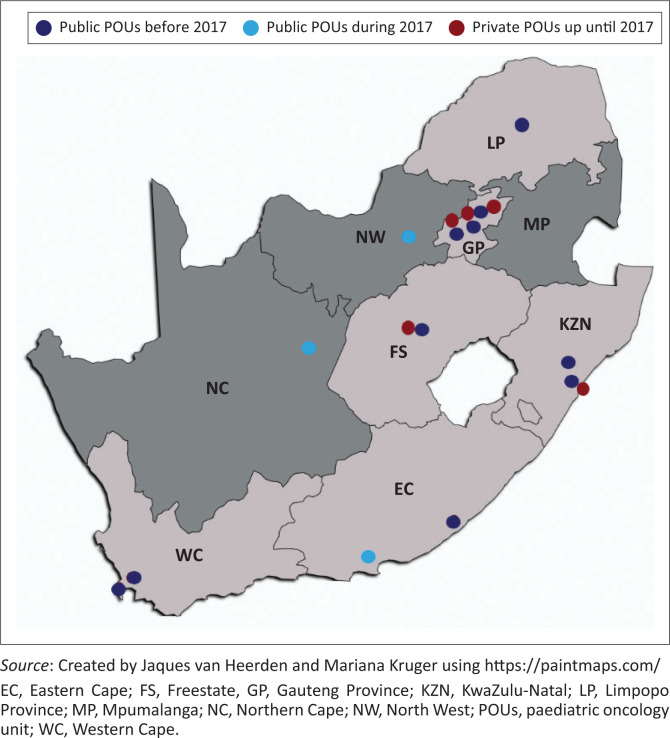
Number of private and public paediatric oncology units in South Africa from 2000 to 2017.

During 2002–2017, private POUs were only located in Bloemfontein, Cape Town, Durban, Johannesburg, and Pretoria (see Figure 2). This was reflected in the provincial distribution between Freestate, Gauteng, KwaZulu-Natal and the Western Cape in the Otoo et al. (2020) study. During the same period, the public sector units have increased from 8 in 2002 to 13 in 2017 (Van Heerden, Esterhuizen & Kruger 2021:1). This further decreased the centralised care and possible private centralised care. It further highlights the inequitable distribution of services that affects whether public or private services can be accessed (Van Heerden et al. 2021:1).

### The age representation

In the Otoo et al. study, the adolescent population represents a disproportionately high percentage compared to international statistics (Steliarova-Foucher et al. 2017). Two possible reasons for this are: (1) adolescents in the private sector (and public sector) are treated by adult oncologists, of whom there are more in the private sector. (2) The greatest percentage of non-adolescent children are treated in the public sector. The very fact of inverse representation indicates a non-representative sample in their data base.

### Increasing populations

The population of South Africa, and thus the child population, has increased in the period described in Otoo et al.’s study. Based on Hall and Sambu’s study, the child population in the country grew by 8%, increasing from 18.1 million in 2002 to 19.6 million in 2017 (Hall & Sambu 2018:132). Therefore, it can be extrapolated that the incidence of childhood cancer over this period should have increased as opposed to the Ooto et al. conclusion of a decrease, even in a limited sector such as private medical aid funding.

### Other funding options

Novel chemotherapeutic options are expensive and not always covered by medical aid funds (Meropal & Schulman 2009:180). To be able to pay for these treatments, families deplete savings, sell property or start funding opportunities such as crowdfunding. The pharmaceutical industry provides medication through compassionate use and medical need programmes (Gerasimov et al. 2020; Moerdler et al. 2019). All of these do not reflect in medical aid payments.

### Possible representative solutions

In South Africa, there are currently two cancer registries that include children and adolescents: The South African Children’s Tumour Registry (SACTR) and the South African National Cancer Registry (SA-NCR). By combining the data from PBMs, SACTR and SA-NCR, while observing the required quality controls for registries data, a truly representative population-based registry can be established.

If PBMs are to be used as a source of data, the research question should be in line with the data possibilities and limitations such as the resources needed during the treatment of children with specific cancers or observing resource trends.

Furthermore, these studies should be conducted per childhood cancer diagnosis as the treatment between malignancies differ vastly.

## Conclusion

Otoo et al. (2020) have presented a very small segment of the paediatric oncology landscape of South Africa while considering the possibility of using the data for policy decisions. The study does not evaluate a representative sample of paediatric oncology care, neither does it recognise the reality of the inequitable paediatric oncology services that exist in South Africa and how it is funded. Therefore, basing policy decisions with far-reaching implications on this study would be flawed.
